# It Is Time to REACT: Opportunities for Digital Mental Health Apps to Reduce Mental Health Disparities in Racially and Ethnically Minoritized Groups

**DOI:** 10.2196/25456

**Published:** 2021-01-26

**Authors:** Elsa A Friis-Healy, Gabriela A Nagy, Scott H Kollins

**Affiliations:** 1 Department of Psychiatry and Behavioral Services Duke University School of Medicine Durham, NC United States; 2 Duke University School of Nursing Durham, NC United States; 3 Duke Clinical Research Institute Durham, NC United States

**Keywords:** digital health, app, public mental health, health disparities, COVID-19, pandemic, mental health, disparity, behavior

## Abstract

The behavioral health toll of the COVID-19 pandemic and systemic racism has directed increased attention to the potential of digital health as a way of improving access to and quality of behavioral health care. However, as the pandemic continues to widen health disparities in racially and ethnically minoritized groups, concerns arise around an increased reliance on digital health technologies exacerbating the digital divide and reinforcing rather than mitigating systemic health inequities in communities of color. As funding for digital mental health continues to surge, we offer five key recommendations on how the field can “REACT” to ensure the development of approaches that increase health equity by increasing real-world evidence, educating consumers and providers, utilizing adaptive interventions to optimize care, creating for diverse populations, and building trust. Recommendations highlight the need to take a strengths-based view when designing for racially and ethnically diverse populations and embracing the potential of digital approaches to address complex challenges.

## Introduction

The dual public health crises of systemic racism and COVID-19 have significantly increased mental health needs while simultaneously limiting access to traditional models of in-person care [1,2]. The pandemic has critically strained an already overburdened system, magnifying existing shortcomings of the mental health system, including inequitable availability, access, and quality of care for the Black, Indigenous, and people of color (BIPOC) community. BIPOC continue to be disproportionately affected (emotionally and physically) by the virus, experiencing more hospitalizations and greater mortality rates as well as increased emotional distress [3,4]. Concurrently, these losses are occurring against a backdrop of inequities due to systemic racism including long-standing disenfranchisement from the health care system, historic traumas, stigma, high cost, and cultural insensitivity, which may put BIPOC communities at highest risk for not receiving mental health care [5,6]. These barriers have persisted in the wake of prior disasters, preventing BIPOC communities from accessing and utilizing mental health treatment after national tragedies [5]. Given the complex and intersecting clinical and social-contextual vulnerabilities that contribute to behavioral health inequities, an urgent need exists for innovative behavioral health approaches that support effective and accessible care to overcome, rather than perpetuate, existing health disparities.

Digital mental health (DMH) tools like mobile apps could play a key role in expanding access to care for those most impacted by COVID-19 and systemic racism by providing remote assessment, support, or intervention; lowering the cost of mental health care; reducing transportation challenges; and providing care in a private and destigmatizing manner [7,8]. However, challenges remain including a lack of culturally grounded DMH interventions, minimal implementation data, low provider and consumer confidence in DMH quality, variability in provider competency in DMH, and lower digital access and literacy in groups at the highest risk for experiencing health care inequities. Digital inequalities span a multidimensional continuum that includes socioeconomic status and location [9], age [10], level of education [11], quality of social support network [12], immigration status [13], location, and health literacy [14]. These inequalities align with social determinants of health [15,16]; therefore, as reliance on digital health approaches increases, digital inequalities may further exacerbate existing health disparities and reduce health care access for those most likely to be affected by the ongoing crises.

In this viewpoint, we first provide a “pulse check” on the DMH field amidst the ongoing pandemic and provide five key corresponding recommendations to ensure optimal leveraging of DMH apps for increasing health equity and ensuring that innovation does not inadvertently widen the digital divide.

## The Current DMH Landscape

Given widespread availability and scalability, DMH apps have been lauded as a potential first line of defense for responding to the increase in behavioral health concerns precipitated by COVID-19 and systemic racism. Recognizing the need to increase access to effective and safe technologies that promote physical distancing, the US Food and Drug Administration (FDA) issued an Enforcement Policy on April 14, 2020, allowing the “…distribution and use of computerized behavioral therapy and other digital health therapeutic devices for psychiatric disorders…” without the requirement that those products comply with traditional regulatory policies. Additionally, the guidance reiterates and clarifies that low-risk wellness and digital health products—including apps that promote mindfulness, meditation, sleep, exercise, or manage mental health symptoms without providing a specific treatment—will continue to be exempt from FDA oversight. This important policy is consistent with the FDA’s previously published emphasis on increasing access to inherently safe digital products for mental health and other medical conditions [17]. FDA guidance regarding mental health apps has the potential to spur innovation, but it can also increase ambiguity in a marketplace already marred with concerns of real-world engagement [18] and a limited evidence base [19,20].

The COVID-19 pandemic has stimulated continued interest and investment in digital health approaches. In the first half of 2020, digital behavioral health startup companies reported record funding of over $588 million, roughly the annual funding for this market in any previous full year, reflecting an industry-wide perception of growing demand for DMH products [21,22]. Continued growth, however, portends continued saturation of the marketplace with apps that make unwarranted claims, offer minimal protection of user data, or are ineffective [23-25]. Health care providers, patients, and their families are at once faced with the promise of leveraging easy-to-access digital tools such as apps, and the daunting challenge of choosing from the 10,000+ DMH apps available [26,27]. The lack of reliable, easily accessible efficacy and safety data around these products continues to impede clinician and consumer ability to make effective health care choices. Reliance on the “wisdom of the crowd” to guide decision making is not advised since, among other reasons, user ratings do not correlate well with clinical utility or quality [27].

## Recommendations

**Figure 1:**
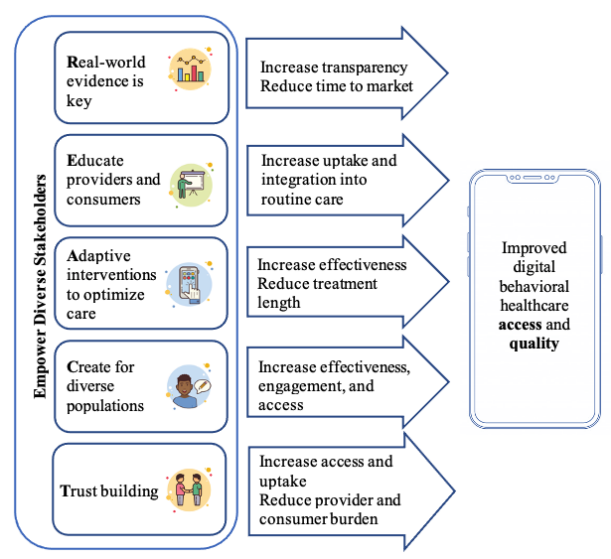
Ecosystem-wide recommendations to increase digital mental health access and quality.

We offer five key recommendations on how the mental health care and technology fields can “REACT” to ensure the development of approaches that increase health equity by increasing *real-world* evidence, *educating* consumers and providers, utilizing *adaptive* interventions to optimize care, *creating* for diverse populations, and building *trust* (Figure 1). As highlighted in Figure 1, these recommendations will only be effective through increased representation of and collaboration with BIPOC researchers, policymakers, educators, providers, developers, and payers, requiring the removal of structural barriers in academia and industry.

### 1. Real-World Evidence Is Key

DMH apps developed for both underrepresented and majority populations have been hindered by a lack of real-world effectiveness data and a paucity of implementation trials [28]. The lack of timely effectiveness data prohibits consumers and medical providers from making informed choices, reducing trust and uptake. Implementation science offers frameworks and methodologies applicable to both increasing sustained use of new technologies and more rapidly evaluating digital products [29]. Drawing on implementation science and human-computer interaction, the Accelerated Creation to Sustainment model [30] offers an alternative approach to traditional clinical evaluation methods that can take over 10 years to move an intervention from conceptualization to implementation [31]. This approach proposes an iterative process of design and evaluation across three phases (create, trial, sustain) and may be facilitated by real-time engagement and outcomes data gathered by the device itself. This approach highlights the need to utilize user-centered design [32,33] to support both the development of a product and an associated implementation and sustainment strategy. In the trial phase, products and implementation strategies are concurrently tested in “real-world” settings utilizing Optimization, Effectiveness, and Implementation studies that explore effectiveness and implementation outcomes. Outcomes such as fidelity, adoption, uptake, and cost are particularly important to track to ensure new technologies are acceptable to a diverse population of consumers. Finally, in the sustainment phase, ongoing passive or low-effort data collection informs continued optimization as the research team transfers responsibilities to clinical or organizational staff.

### 2. Educate Providers and Consumers

As the field moves toward generating a more robust and easily interpretable evidence base for specific apps, providers and consumers must be empowered with alternative strategies to make informed decisions on which apps to utilize in clinical practice. The American Psychiatric Association’s app evaluation framework offers a guiding framework for providers and consumers to self-evaluate apps [34]. In principle, this framework offers a much-needed solution to a complex problem, but it also places a potentially high burden on providers to evaluate digital therapies, which the framework itself notes is “not what psychiatrists and mental health clinicians are classically trained to do [provide]” [35]. Given these challenges, it is unclear how provider evaluation of mental health apps will practically fit into the workflow for clinicians.

Moreover, given that a substantial proportion of mental health services is rendered through primary care [36], additional steps need to be taken to help provide primary care providers with the necessary information to make informed decisions about app selection for their patients. It is therefore critical to train both primary care and mental health professionals in digital technologies to support patient care. Frameworks for digital competencies in mental health have primarily focused on telehealth and do not fully address integration of broader products including the prescription of digital therapeutics or integration of apps to support treatment [37,38]. There must be a synergistic balance between providers being empowered to effectively choose and evaluate digital products on an individual basis for their patients, and more transparent and accessible information on individual products. The balance may be facilitated by the inclusion of a digital specialist or “digital navigator” [39]. Finally, broader provider and stakeholder education is critical as lack of understanding or knowledge of digital approaches is a key barrier to DMH uptake and adoption [40].

### 3. Adaptive Interventions to Optimize Care

Digital approaches offer the opportunity to develop adaptable interventions that provide tailored content based on individual characteristics, such as race, gender, sexual identities, family structure, and language preferences [41]. The pandemic has further highlighted the potential for external stressors to exacerbate previously managed chronic mental health concerns and the need to flexibly ramp up care. We can leverage novel clinical trial methodologies (eg, the Multiphase Optimization Strategy [42] and sequential, multiple assignment, randomized trials (SMART) [43]) to develop adaptive DMH interventions that adjust the type or dosage of the intervention based on patient characteristics. SMART trials facilitate this development by randomizing patients to different treatment options across time based on predetermined decision points, allowing for an assessment of effectiveness for different interventions at each stage. These novel designs allow for the evaluation of the tailoring variables and intervention components in the same trial. Moreover, they support the development of decision rules for assigning treatments based on patient characteristics and response to interventions, rather than a priori decisions. This can result in shorter and more targeted interventions.

### 4. Create DMH Apps for a Diverse Population of Users

Considerable work is required to ensure the availability of DMH products that fit a wide range of user needs and preferences. Efforts have been hampered by the underrepresentation of BIPOC researchers and developers, calling for fundamental shifts to address structural barriers in academics and industry. In order for digital technologies to recognize their full potential to mitigate widening behavioral health disparities exacerbated by the COVID-19 pandemic and ongoing cultural-based traumas, they must be designed with those who are both at most risk for mental health concerns and who face the greatest barriers to health care engagement. At risk of oversimplification, this fundamentally calls for us to follow the core principle of designing for the end user [32]. This includes both the type and way we utilize technology to deliver care and the intervention content we deliver. Human- or user-centered design approaches that ask the question, “What are the barriers to receiving effective behavioral health care and how can technology help overcome them?” must be used to develop strategies that mitigate disparities. However, such approaches must engage consumers whose voices are traditionally underrepresented in care settings. Culturally salient recruitment strategies [44], which include addressing community mistrust, participant resource constraints, and potential risks (eg, community stigma), could help researchers and developers more effectively integrate the input of those consumers most likely to benefit from their products [45].

BIPOC underrepresentation in the product development process has potentially reinforced structural inequalities in our health care system by limiting the availability of products that are culturally inclusive and effective [8]. The lack of culturally appropriate tailoring, stakeholder input, and broader community-partnered implementation plans limits the effectiveness of DMH tools, leads to decreased uptake, and may contribute to lower rates of DMH utilization in BIPOC populations despite equal or higher preferences for using DMH apps compared to White peers [46,47]. To increase potential effectiveness and uptake, cultural tailoring must go beyond language translation to incorporate cultural values, norms, and references [48]. This tailoring offers the opportunity to capitalize on community or cultural resiliency factors offering opportunities to increase efficacy of interventions in addition to improved engagement. Finally, digital technologies offer the opportunity to develop intervention approaches that are not as inherently embedded in cultural paradigms or at minimum are language agnostic (eg, attentional bias modification) [49].

### 5. Trust Building Through Monitoring and Independent Vetting

As we work toward the development and rapid real-world evaluation of DMH approaches for diverse populations, there is a strong need for a sustainable ecosystem to increase consumer and provider confidence, and empower users and providers to effectively choose appropriate and effective DMH interventions based on their specific needs and context. At an aspirational level, there are several approaches that could help increase this confidence.

### Increase Industry and Marketplace Self-Monitoring

Across all DMH products, there must be increased industry pressure to self-monitor, conduct rigorous research, and disseminate results in a manner that is interpretable by both consumers and providers. A potential short-term step toward this end is clear reporting of digital products released under the COVID-19–related FDA guidance to ensure company accountability to consumers and facilitate uptake of new low-risk products. On the open app marketplace, the inclusion of clear, easily interpretable statements regarding potential efficacy and risks, including data security, must be encouraged. A review of 73 popular apps found that none of these evaluations or membership in app libraries were noted in their app store descriptions [27], suggesting a potential lack of awareness of these schemes outside academic communities or that accreditation could benefit companies. App stores could therefore provide a more standardized way to include reporting of clinical testing results [50]. It is recognized that these recommendations introduce burden to companies and, without enforcement-related contingencies, are unlikely to be fully effective. Nevertheless, continued dialogue among stakeholders, including patient and advocacy groups, providers, and DMH companies, will help advance the field.

### Independent Vetting and Structured Dissemination of DMH Products

The lack of reliable, easily accessible efficacy and safety data impedes clinician and consumer ability to make effective health care choices. Digital formularies that provide a curated list of clinically supported DMH products could be an important mechanism for enabling dissemination of products including apps into clinical care [51]. Formularies could integrate into electronic health records and associated patient portals, allowing both patients and providers to know which products are supported by their health care organization. Indeed, some steps have been taken in this regard, and lessons learned from these efforts will be important to refine the approach [52,53]. Similarly, the development of independently curated app libraries [54,55] takes a clear step toward increasing transparency around the safety and potential efficacy of apps outside of a formalized health system. However, attempts are hindered by the vast quantity and rapidly changing nature of products on the market. Conscious of the challenges of keeping up with this massively expanding and growing field, the FDA’s Digital Health Precertification Pilot Program [56] shifts regulations toward the app developers and manufactures themselves, allowing for products to be developed efficiently through rapid iteration more appropriate for digital products, therefore reducing the need for burdensome hurdles to be cleared with frequent software changes, etc. For this to be successful, there must be an understanding between regulators and developers of a consensus framework for product evaluation. In addition, there must be adequate incentives for engagement in any regulatory programs, as prior voluntary certification attempts have failed [57].

## Conclusion

COVID-19 and increased awareness of systemic racism have highlighted the promises and pitfalls of DMH. As interest in DMH grows, we have the opportunity and responsibility to increase the availability of safe, effective, and accessible products that reduce rather than perpetuate health disparities. This will require synergistic collaboration among researchers, policymakers, educators, providers, developers, payers, and patients. As researchers and clinicians, we are well positioned to lead this charge; however, we must embrace the rapid nature of technological innovation and development. Concerns around the increasing digital divide must serve as a “wake up call” for the DMH field to ensure products and mHealth (mobile health) approaches are tackling health care disparities rather than contributing to them. Rather than “designing down” to reduce the complexity of interventions for populations with more limited digital literacy, we must take a strengths-based view of designing for diverse populations [8]. By embracing these strengths, digital health has the opportunity to increase access to care for the most vulnerable populations. Finally, fundamental shifts to address structural inequalities in academics and industry, including the underrepresentation of BIPOC researchers and developers, are needed to revolutionize our field.

## Abbreviations

BIPOC: Black, Indigenous, and people of color
DMH: digital mental health
FDA: Food and Drug Administration
mHealth: mobile health
SMART: sequential, multiple assignment, randomized trials
